# P-1820. Clinical Factors and Obstetric Outcomes of Pregnant Women with Hepatitis C in Southeast Michigan

**DOI:** 10.1093/ofid/ofaf695.1989

**Published:** 2026-01-11

**Authors:** Angela Ishak, Yasmeen Mann, Johnny Zakhour, Brianna Hohmann, Indira Brar, Geehan Suleyman

**Affiliations:** Henry Ford Hospital, Detroit, MI; Henry Ford Hospital, Detroit, MI; Henry Ford Health, Detroit, Michigan; Henry Ford Health, Detroit, Michigan; Henry Ford Hospital, Detroit, MI; Henry Ford Health, Detroit, Michigan

## Abstract

**Background:**

The prevalence of maternal hepatitis C virus (HCV) infection increased 16-fold from 1998 to 2018, potentially due to high rates of intravenous drug use among women. Data suggest that pregnant women with detectable HCV viral loads (VL) are more likely to experience pregnancy complications such as preterm delivery and fetal growth restriction. However, literature on the risk factors and obstetric outcomes in pregnant individuals with HCV infection remains limited.

**Figure:**
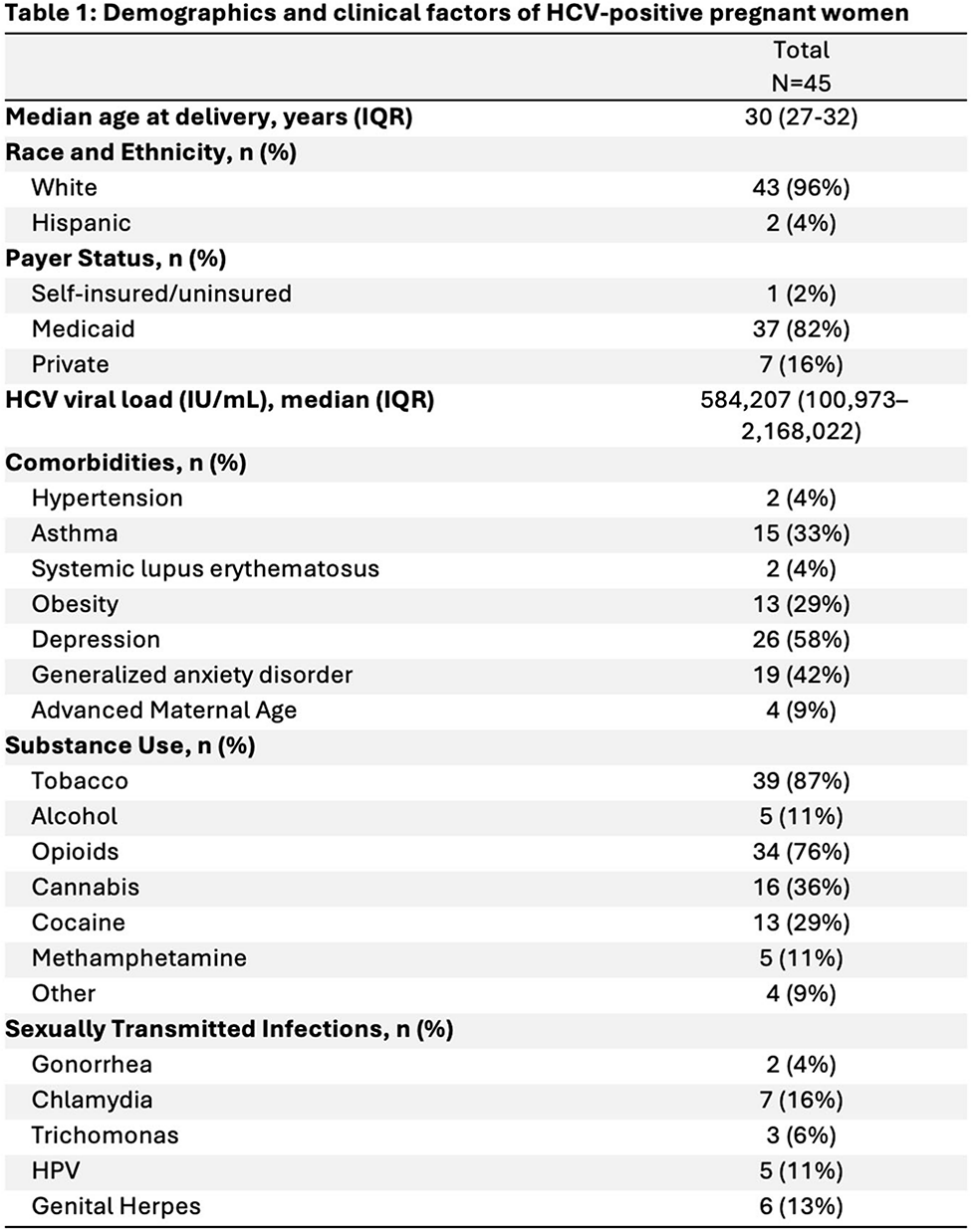

**Figure:**
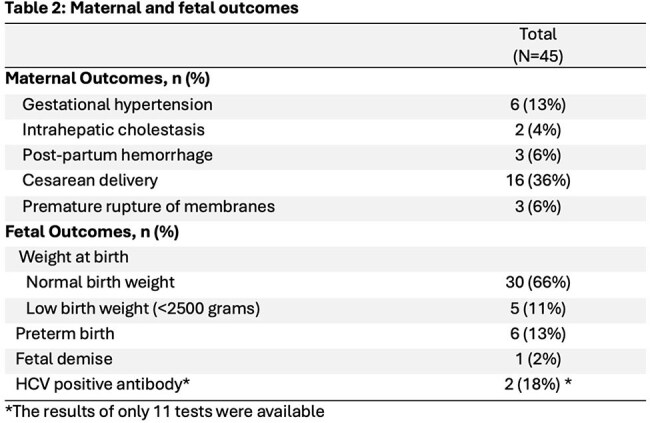

**Methods:**

This is an observational study of pregnant women aged ≥18 with HCV who delivered at Henry Ford Health in Southeast Michigan from 2013–2024. Demographic data, clinical factors (including comorbidities, sexually transmitted infections [STIs], substance use), and maternal and fetal outcomes were evaluated. Low birth weight was defined as < 2,500 grams. Maternal and fetal outcomes among viremic and non-viremic women were compared.

**Figure:**
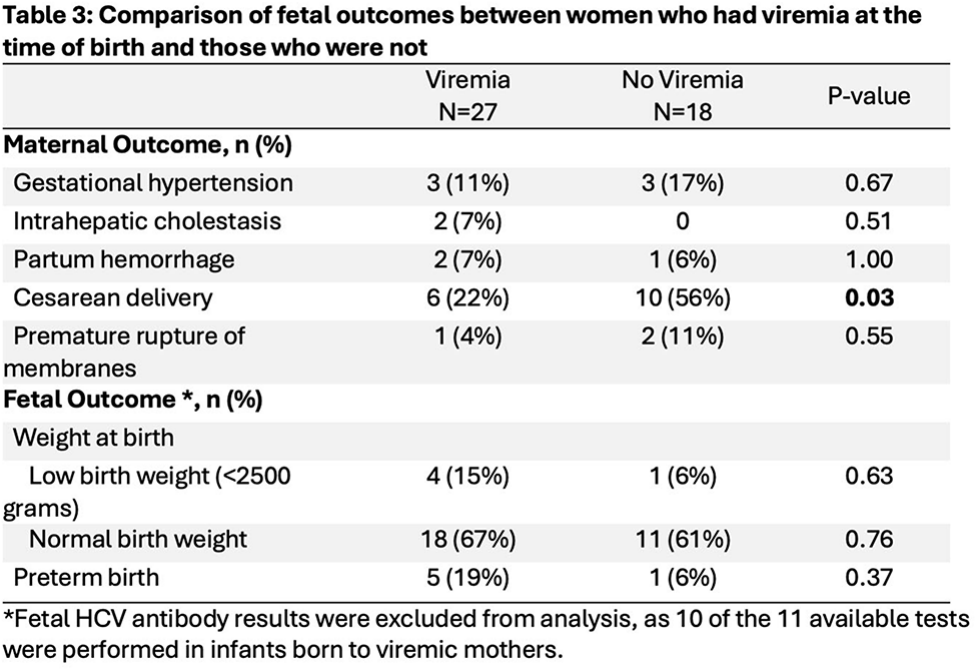

**Results:**

Among 67,534 women who delivered, 45 (0.07%) had HCV with median VL of 584,207 (Table 1). Of these, 27 (60%) were viremic at delivery. Most were white (96%) and publicly insured with Medicaid (82%). High rates of substance use were noted. Common comorbidities included depression (58%), anxiety disorders (42%), and asthma (33%); STIs were common but no patients had HIV. Maternal outcomes included need for cesarean delivery (36%), gestational hypertension (13%), postpartum hemorrhage (6%), and premature rupture of membranes (6%) (Table 2). Most infants had normal birth weight (66%); preterm birth (13%) and low birth weight (11%) were not uncommon. Of the 11 neonates tested for HCV antibody, 2 (18%) were positive. Regarding maternal and fetal outcomes, rate of cesarean delivery was significantly different between the groups (55.6% vs 22.2%, p=0.03) (Table 3).

**Conclusion:**

HCV-positive pregnant women in our cohort had high rates of opioid use, mental health conditions, and adverse perinatal outcomes. Viremia at delivery was not associated with worse maternal or fetal outcomes, except for cesarean delivery. These findings underscore the importance of universal HCV screening with each pregnancy and the role of preconception diagnosis and management of HCV infection. Early identification allows for treatment initiation prior to pregnancy to improve maternal and fetal outcomes.

**Disclosures:**

Indira Brar, MD, Gilead: Advisor/Consultant|Gilead: Grant/Research Support|Gilead: Honoraria|ViiV Healthcare: Advisor/Consultant|ViiV Healthcare: Grant/Research Support|ViiV Healthcare: Honoraria

